# ResBCDU-Net: A Deep Learning Framework for Lung CT Image Segmentation

**DOI:** 10.3390/s21010268

**Published:** 2021-01-03

**Authors:** Yeganeh Jalali, Mansoor Fateh, Mohsen Rezvani, Vahid Abolghasemi, Mohammad Hossein Anisi

**Affiliations:** 1 Faculty of Computer Engineering, Shahrood University of Technology, Shahrood 3619995161, Iran; jalali.yegane@gmail.com (Y.J.); mrezvani@shahroodut.ac.ir (M.R.); 2 School of Computer Science and Electronic Engineering, University of Essex, Colchester CO4 3SQ, UK; m.anisi@essex.ac.uk

**Keywords:** segmentation, lung, CT image, U-Net, ResNet-34, BConvLSTM

## Abstract

Lung CT image segmentation is a key process in many applications such as lung cancer detection. It is considered a challenging problem due to existing similar image densities in the pulmonary structures, different types of scanners, and scanning protocols. Most of the current semi-automatic segmentation methods rely on human factors therefore it might suffer from lack of accuracy. Another shortcoming of these methods is their high false-positive rate. In recent years, several approaches, based on a deep learning framework, have been effectively applied in medical image segmentation. Among existing deep neural networks, the U-Net has provided great success in this field. In this paper, we propose a deep neural network architecture to perform an automatic lung CT image segmentation process. In the proposed method, several extensive preprocessing techniques are applied to raw CT images. Then, ground truths corresponding to these images are extracted via some morphological operations and manual reforms. Finally, all the prepared images with the corresponding ground truth are fed into a modified U-Net in which the encoder is replaced with a pre-trained ResNet-34 network (referred to as Res BCDU-Net). In the architecture, we employ BConvLSTM (Bidirectional Convolutional Long Short-term Memory)as an advanced integrator module instead of simple traditional concatenators. This is to merge the extracted feature maps of the corresponding contracting path into the previous expansion of the up-convolutional layer. Finally, a densely connected convolutional layer is utilized for the contracting path. The results of our extensive experiments on lung CT images (LIDC-IDRI database) confirm the effectiveness of the proposed method where a dice coefficient index of 97.31% is achieved.

## 1. Introduction

Lung cancer is known as the second most prevalent type of cancers in both genders in the world [1]. According to the World Health Organization (WHO), lung cancer is responsible for 1.3 million deaths per year in the world [2]. It is estimated that around 228,820 new lung cancer cases (116,300 in men and 112,520 in women) and around 135,720 deaths from this disease (72,500 in men and 63,220 in women) are identified in the United States each year [3]. Lung cancer is known as a malignant tumor characterized by the unnatural growth of the cell in the lung tissue. Rapid diagnosis of this cancer can significantly decrease the death rate and enhance patient survival chances. This is very important in improving the clinical situation of patients. Thus, it is necessary to present an intelligent algorithm for the early diagnosing of lung cancer.

Recent advances in computer vision and image processing technologies have significantly helped the healthcare systems particularly in the analysis of medical images. In this regard, image segmentation is widely used as one of the most fundamental, useful, and well-studied topics in image analysis. Image segmentation can significantly improve the recognizability of parts of an image by assigning a label to each pixel in the image such that those pixels with the same labels have similar visual features characteristics.

Segmentation is a substantial process in medical image processing and can reveal very useful information concealed in the images. In some medical applications, the classification of image pixels into descriptive regions, such as bones and blood vessels, is of interest. While in other applications it is more appropriate to look for pathological regions, such as cancer or tissue deformities [4]. One of the most important segmentation tasks in medical images is to identify redundant pixels or unwanted regions located as background. This segmentation is considered as one of the most challenging steps, especially in CT (computed tomography) or MRI (magnetic resonance imaging), to provide critical information about the shapes and volume of body organs. In other words, the overall performance of automated cancer detection is highly dependent on the output of the segmentation stage [5].

**Figure 1:**
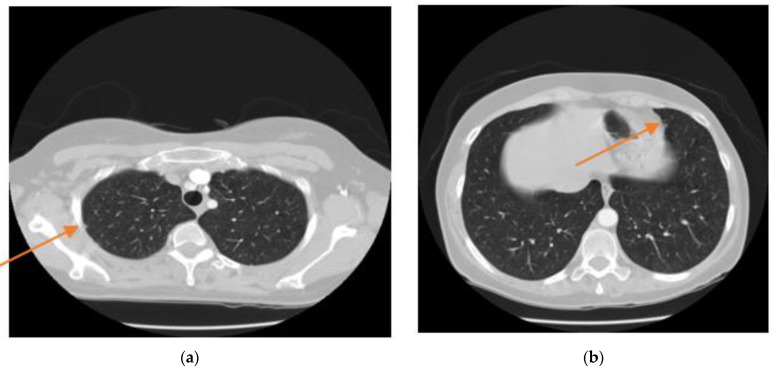
Two examples of nodules attached to the lung wall in CT-scan images. (**a**). represents one nodule attaching to the outer wall of the lung, (**b**). represents one nodule attaching to the outer wall of the lung (orange arrows).

In the lung segmentation stage, we seek to distinguish those pixels associated with the lung from every other pixel in the surrounding anatomy. Radiologists often use a CAD (computer-aided design) system to provide a secondary consideration for an accurate diagnosis. This method is useful for improving the efficacy of the cure. For many CAD systems, a precise segmentation process of the target organ is required, which is a fundamental step and a prerequisite for effective image analysis. The segmentation of lung fields is particularly challenging because the lung zone is highly inhomogeneous. In addition, pulmonary structures present similar congestions in different scanners and scanning protocols which make the segmentation difficult. It becomes even more challenging because of the presence of nodules attached closely to the lung wall. Figure 1 offers two examples of lung CT scans that show the exact location of the node attached to the lung wall. This figure also clearly represents the challenge of dividing the lungs despite these nodules.

Medical image segmentation is an important and inseparable step in the diagnosis process. For example, in the process of diagnosing lung cancer, the main steps are as follows: (1) image pre-processing; (2) image segmentation; (3) feature extraction; (4) lung cancer identification; (5) diagnosis of the disease [6,7]. It so happens that various algorithms directly use the segmentation step in their work [8,9,10]. For example, Wang et al. [10] conducted a study on differentiating COVID-19 from non-COVID-19 CT scans. In their proposed method, images of patients were first segmented during a single step using a deep neural network. Then, the images and tags were given to a network for classification. They could achieve a 0.959 ROC AUC score. Unlike the previous example, some methods extract the region of interest and do segmentation indirectly within the feature extraction stage [11,12,13]. For example, Pathak et al. [13] proposed a system for the detection of COVID-19 in CT scans that considered a prepressed transfer learning. The system used a neural network to extract the features from CT images, and a 2D convolutional neural network was considered for the classification. The proposed system was tested on 413 COVID-19 and 439 non-COVID19 images with 10-fold cross-validation, and it achieved 93.01% accuracy.

It is clear that medical image segmentation is always accompanied by disease detection algorithms. However, algorithms that specifically try to segment with high accuracy will ultimately perform better for the diagnostic model. For this reason, we will also present a robust system for accurate segmentation of the lung area in this article.

Generally, many techniques have been reported in the literature for the segmentation of medical images. The most important drawback of the existing methods is relying on the utilization of manual (hand-crafted) features to successfully segment the regions of interest. In addition, most techniques are unable to segment nodules attached to the lung wall. Recent advances in medical image processing by using deep learning-based methods have revealed great influences in clinical applications. These methods can appropriately learn important features of medical images and consequently overcome the limitation of hand-crafted features [14]. In this paper, we propose a deep learning-based method to accurately segment the lung tissue. In order to achieve a successful segmentation, we require the raw CT images with their associated ground truths. Unfortunately, current lung CT databases do not come with binary masks (ground truths). Hence, we propose a semi-automatic method to resolve this issue by producing the corresponding masks. Then, we apply appropriate pre-processing steps in order to enhance the quality of images used in the training phase. In the last phase, all these pre-processed images with corresponding binary masks are fed into a deep neural network. Our proposed deep model is a combination of the ResNet and BCDU-Net. In fact, the backbone and the basis of the deep learning network used in this paper are BCDU-Net. On the other hand, using pre-trained networks such as ResNet, which have been trained in the ImageNet data collection, increases the speed of training and the power of the network extension. So, the proposed method in this paper is a novel BCDU-Net architecture that takes the advantage of ResNet-34 instead of ordinary convolution layers in the encoding section.

The contributions of the current manuscript are:

- Applying novel extensive preprocessing techniques to improve quality of the raw images.
- Proposing a new method for extracting ground truths corresponding to the input images.
- Employing a new deep learning-based algorithm for proper segmentation of lungs.

The rest of this paper is organized as follows: Section 2 reviews some previous segmentation models. Section 3 introduces the proposed method in detail. Section 4 is devoted to evaluating the performance of our method through extensive experiments. Section 5 draws some conclusions. Section 6 highlights future works.

## 2. Related Works

There are several techniques that have been developed to address the segmentation task. Most of these approaches are mainly divided into five categories: threshold-based, edge-detection, region growing, deformable boundary, and learning-based methods. In what follows, we briefly review these categories.

### 2.1. Threshold-Based Methods

Since the lungs are filled with air during the CT scan, they are characterized by dark areas in the associated grayscale image. Therefore, threshold-based approaches rely on this principle that normal lung tissues have less density than the surrounding regions. On this basis, the lung regions are separated by specifying a suitable threshold on the images [15]. These approaches are of the most popular lung segmentation methods because of their simplicity in performance and computation. They can also be used in real-time applications. However, these methods have some deficiencies in lung segmentation. (1) They are not able to effectively remove the trachea and main stem bronchi [16]. (2) Due to various conditions in different images like air volume and image acquisition protocol, a universal gray-level segmentation threshold would not be suitable [17]. (3) They are not often successful in cases where anomalies represent higher densities compared to those in natural lung tissues [18].

### 2.2. Edge-Detection Methods

Lung segmentation can be also performed by using edge detection techniques. Edge in image processing is defined as the boundary between the two regions with relatively distinct gray surface properties. Some of the well-established spatial edge detection techniques are Prewitt, Robert, Sobel, Prewitt, Laplacian, and Canny. In what follows, we refer to canny as the most effective edge detector algorithm.

Canny is a well-known conventional edge detection algorithm. It can find the edges of image regions by isolating noise from the image. The main advantage of this method is that it does not affect the properties of the image edges and find edges and critical thresholds. Canny is capable of achieving three important properties, i.e., great localization of edge points, small error rate, and one-to-one responses to every single edge. As a result, it normally performs well, thus, it is considered as one of the best methods to extract the edges compared to other existing methods [19]. Shin et al. [20] demonstrated the performance evaluation of different edge detectors and concluded that the Canny detector has the best performance and robustness compared to other edge detectors. In this regard, Campadelli et al. [21] detected edges from chest radiograph images and achieved an accuracy of 94.37%. Mendonca et al. [22] identified the image edges using a spatial detector for lung tissue segmentation in radiograph images. They used 47 radiograph images and achieved a sensitivity of 0.9225 and a positive predictive value of 0.968.

In brief, the benefits of edge-based methods are (1) performing well in discriminating between the background and the objects within an image, (2) high-level approach in image segmentation similar to the way human perception segments the images. The main deficiencies of these methods are: (1) sensitivity to noise, (2) working inappropriately on images with smooth transitions and low contrast.

### 2.3. Region Growing Methods

Segmentations based on image regions are called region growing techniques. The basic idea in this method is to collect pixels posing similar characteristics within a commonly formed area. In another word, this category of methods starts the segmentation process with a set of seeds. The seeds in any given image, can either be one single pixel or a group of several pixels. After forming the seeds, the next step is to determine whether the neighboring pixels must be added to the region or not. This is decided based on similarity criteria such as color, intensity, variance, texture, and motion. Gradually, these pixels begin to grow and form regions. Finally, when the image is completely divided by all the growing regions and all the textural stages of the image are obtained as the boundaries of the final regions, the algorithm is terminated. Region growing methods are utilized in many medical applications such as cavities segmentation in the cardiac images [23], blood vessel extraction in the angiographic data [24], renal segmentation [25], brain surface extraction [26], and lung CT image segmentation [27].

Region growing technique has some advantages including low computational complexity and high speed. However, its performance is highly dependent on the location of the seed points and the growing conditions. It can be stated that region growing methods are sensitive to noise or variation of intensity. This could result in holes or over-segmentation and also dependency performance on its initial seeds. Its particular disadvantage in lung CT images is that it cannot segment the nodules attached to the borders of the lung image [13].

### 2.4. Deformable Boundary Models

These models consider the entire object’s boundary and can incorporate prior knowledge about the object’s shape as a constraint toward a precise segmentation outcome. For example, in lung segmentation, the boundary of the lung is determined by the evolution of particular interior and exterior forces to fit the shape of the lung. Therefore, the parametric representations used in these models can provide a concise and analytical description of the lung. The most popular approach in deformable models is an active contour model or snake [28]. Itai et al. [29] segmented the lung region from a CT image using a 2D parametric deformable model, called the SNAKES algorithm, without considering any manual operations. Shi et al. [30] proposed an extraction technique for the lung region by using a new deformable model through radiograph images.

Also, there exist some active contour models with many privileges such as providing smooth and closed segmented contours and obtaining sub-pixel details of the object’s boundaries [14]. However, one of the limitations of these models is that they often require human interaction within the construction of the initial contour. Therefore, they normally perform poorly in non-interactive applications, as the algorithm cannot be initialized close to the desired structure of interest. Another limitation of the SNAKE model is that they have weak convergence in the face of boundary concavities.

### 2.5. Learning-Based Models

Learning-based approaches are presented in the area of segmentation of medical images as well. In traditional learning-based methods, the segmentation process is addressed as engineered features. Pixel classification-based approach [31] is known as one of the most important categories in these techniques. However, it is very challenging to select sub-pixels and extract some features to train the classification of a greater number of pixels. To overcome this problem a super pixel learning-based method have used in [32] to prune the pixels and merge them with the confined regions of shape constraints to segment lung CT images. Generally, these methods have two shortcomings to extract the features. The first drawback is relying on using hand-crafted features to achieve the segmentation results. Another limitation is that designing the representative features for different applications is very difficult.

Segmentation techniques based on deep learning can be ranked as pixel-based learning techniques for classification. Unlike conventional pixel or super-pixel classification methods, which often use hand-crafted features, deep learning approaches can process natural data in its raw form as well as learning features and overcoming the limitations of hand-crafted features [19]. These approaches have predominately utilized for semantic segmentation of natural image scenes and have also found many applications in biomedical image segmentation tasks. They also contributed to decrease the manual manipulations needed for segmentation and improving the accuracy and speed of segmentation. One of the most important recent applications of segmentation is to accurately quantify the COVID-19 virus effects. In [33], a new deep-learning-based method is used for automatic screening of COVID-19 with limited samples in order to complete the screening of COVID-19 and prevent further spread of the virus.

Previous deep learning methods purposed for medical image segmentation are mostly based on the patches of images. Convolutional neural network (CNN) is the most successful and widely used approach among many deep learning architectures community for medical image analysis [34]. It is easy to use CNN to classify each pixel in the image separately by offering the extracted neighboring regions of a particular pixel. For example, the authors in [35] proposed a method based on light patches and sliding windows neuronal membranes segmentation in microscopic images. This method has two deficiencies: redundant computation caused from sliding window and huge overlap within input patches from neighbor pixels.

**Figure 2:**
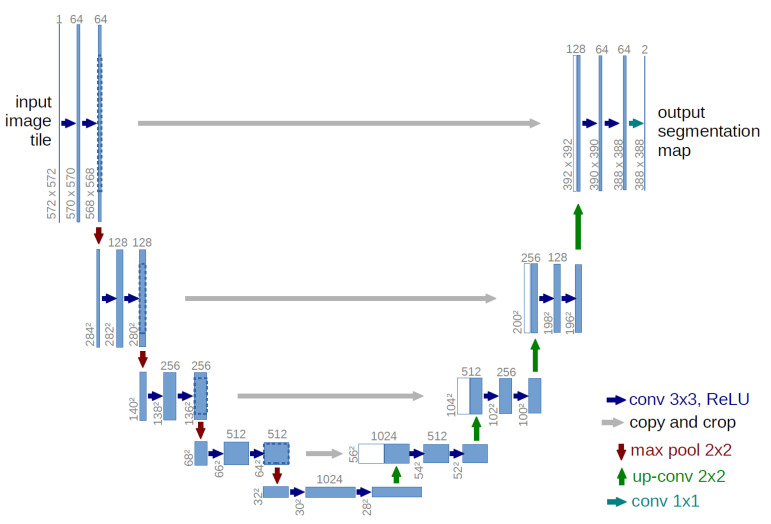
The U-Net architecture [37]. In the contraction path of this network, feature channels are doubled in each down-sampling. Conversely, the expansion path is responsible for decreasing feature channels. The skip connections are also displayed with gray arrows drawn to incorporate two feature maps.

To overcome these problems, the use of a fully convolutional network (FCN) was introduced by Long et al. [36] in which the last fully connected layers of the CNN replaced by transpose convolutional layers. With emerging of the end-to-end FCN, Ronneberger et al. [37], using the idea of the FCN, proposed U-shape Net (U-Net) framework for biomedical image segmentation. U-Net is one of the most popular FCNs for segmentation of medical images. U-Net configuration (Figure 2) comprises two paths; a contracting path to capture context and a symmetric expanding path to obtain accurate localization. The contraction path includes consecutive convolutional layers and max-pooling layer. It is used to extract attributes while constraining the attributes map size. The expansion path achieves up-conversion and has the convolution layers to retrieve the size of the feature maps with the loss of localization knowledge. Also, the localization information is shared from the contraction layer to the expansion layer by applying skip connections. These connections are utilized in parallel and allows data to be transmitted directly from a network block to another with no extra computational cost. Ultimately, the convolution layer draws the attribute vector to the number of classes required at the final partitioning output. The U-Net model has some advantages compared to other patch-based segmentation approaches [38]: (1) It works well with very few training data. (2) It can utilize the global location and context information simultaneously. (3) It ensures maintenance of the complete texture of the input images.

U-Net has offered state-of-the-art performance in biomedical image segmentation. In recent years, different extensions of U-Net have been proposed [39,40,41,42,43]. For example, Milletari et al. [39] proposed V-Net as an extension of U-Net for 3D medical image segmentation. Furthermore, in an extended paper, Cicek [40] proposed a U-Net architecture for 3D images. Zhou et al. [41] developed a nested U-Net architecture. Other researchers have developed various extensions of the U-net. The most significant changes in these methods are mainly related to the skip connections. For example, in Attention U-Net [42], the extracted features at the skip connection are transferred to a processing stage first, and then they are concatenated to each other. One of the limitations of these networks is their two-stage process, i.e., first applying separate processing steps to each group feature map and then concatenating the feature maps together. In [43], a residual attention U-Net was proposed for automated segmentation of COVID-19 Chest CT images. This deep learning model is based on U-Net which uses the residual network and attention mechanism to enhance feature extraction and generate high-quality multi-class segmentation results. The use of this method has led to 10% improvement in the segmentation performance.

In order to improve the original U-Net network, instead of using the desired convolution layers, various other architectures can be used in the encoding part of this network. For example, a U-Net-based network is presented in [44] wherein the ResNet34 pre-training model is used in its contraction path (left U). The greatest advantage of this modification is increasing the speed of training and the power of the network extension.

In another work, U-Net has been extended to a network called BCDU-Net [45] and achieved better performance than modern alternatives for medical image segmentation. In this network, the encoding path includes four stages. Each stage is composed of two 3 × 3 convolutional filters on the image. After each convolution filter, there is a 2 × 2 max-pooling and a RELU activator. These three layers together form a down-sampling process. In each down-sampling, feature channels are doubled. The encoding path gradually extracts the representation of images and increases the dimensions of the representation layer by layer. This network offers two contributions. First, it uses densely connected convolutions to prevent the learning redundant features problem in successive convolutions in the last encoding path layer of general U-Net. Second, batch normalization is utilized in the decoding path after each up-sampling stage. Batch normalization helps to improve the performance, speed, and stability of neural networks. The resulting output from the batch normalization function is given to a bidirectional convolutional LSTM [46] (BConvLSTM). The feature maps are processed with BConvLSTM to integrate in a more complex way than simple concatenation in U-Net. BConvLSTM itself applies two ConvLSTMs on the input data in both forward and backward directions and then determines the data dependencies in both directions.

According to the above discussions and also the pre-trained ResNet framework [47] that makes the neural network wider, deeper, and faster, we propose an architecture that is mainly inspired by BCDU-Net and ResNet34 to automatically segment the lung CT images. In the next section, the proposed model will be described and presented with all the required details.

## 3. Proposed Method

**Figure 3:**
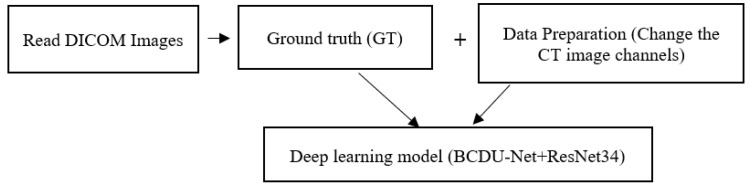
The pipeline of the proposed method.

The proposed model encompasses three major steps: (1) ground truth extraction, (2) image pre-processing and data preparation, and (3) deep learning-based segmentation. Moreover, our novel deep learning model is composed of BCDU-Net and ResNet34. The block-diagram of different steps of the proposed method is depicted in Figure 3. In what follows, we first introduce the database used in this study followed by a description of the process of semi-automatically re-producing database images. Then, we provide pre-processing operations to prepare data stepwise. Finally, we describe the method based on deep learning to segment these images and the corresponding masks.

### 3.1. DICOM Images Reading

In this paper, we used the LIDC-IDRI dataset which involves lung cancer CT scans with marked-up annotated lesions as well as diagnostic information [48]. It is an internationally available resource of development, training, and assessment of diagnostic methods used by the computer (CAD) to diagnose lung cancer. All CT scans are in DICOM format and measured in HU and they have three channels and a resolution of 512 × 512. The original DICOM images and their corresponding XML files are related to 1018 CT scans of 1010 patients registered in this data collection. These images consist of a chest CT scan and an XML file annotated by four professional medical experts. The first step is to read and import these DICOM images.

### 3.2. Ground Truth (GT) Extraction

Our deep learning architecture requires both input images and their corresponding ground truth for successful segmentation. This database lacks labels for lung images, thus, we need to manually extract every ground truth for CT images. Ground truth is in form of masks that could be used to extract ROI from images to be then fed to the deep learning model. Because the ground truth plays a vital role in the segmentation process, custom masks were created using a semi-automatic technique so that they could be verified to be ‘correct’.

**Figure 4:**
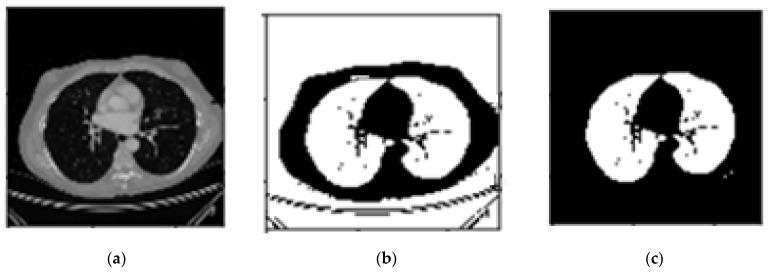
(**a**). Main CT image, (**b**). Binary image, (**c**). Image after eliminating border blobs.

In the CT scans, the lungs are declared as dark zones, while lighter areas inside the lungs are considered to be blood vessels or air. The purpose of this step is to extract lung regions as accurately as possible from each CT scans slice. This step should be performed with extra care to avoid missing any region of interest particularly those attached to the lung wall. Seven steps are carried out to get the masked lungs. These are as follows [22]:

1. Conversion to binary image: In the first step, slices of DICOM images are converted into binary using the threshold method represented by Equation (1). A threshold of -604 HU was applied to extract lung parenchyma [23]. The transformed image to binary is shown in Figure 4b.

(1) $$Binary\;\left(i,j\right)=\{\begin{array}{c} 1\;\;\;\;\;if\;\;\;\;\;f\left(i,j\right)<T\\ 0\;\;\;\;\;\;\;\;\;\;\;\;\;\mathrm{otherwise} \end{array}\;\;\;\;\;\;\;,\;\;\;\;\;T=604$$

**Figure 5:**
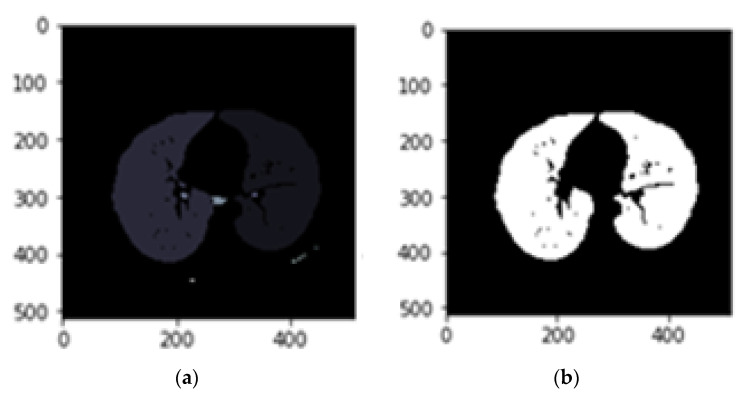
(**a**). Labeled image, (**b**). Image with the two largest labeled areas kept.

**Figure 6:**
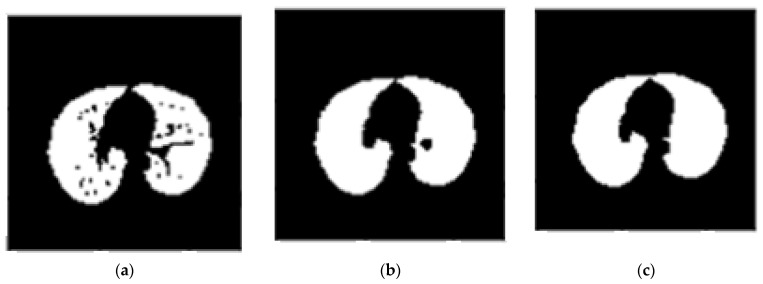
Results of applying (**a**). Erosion operation, (**b**). Closure operation, (**c**). Filling small holes (binary mask).

2. Removing the blobs connected to the CT image border: To classify the images correctly, the regions connected to the image border are removed, as shown in Figure 4c.

3. Labelling the image: Pixel neighbourhoods with the same intensity level can consider being a connected region. When this process is applied to the entire image some connected regions are formed. Figure 4a shows connected regions of integer array of the images that are labelled.
4. Keeping the labels with two largest areas: As shown in Figure 5b, labels with the two largest areas (both lungs) are kept whereas the tissues with areas less than the expected lungs are removed.

5. Applying erosion operation (with a disk of radius 2): This operation is applied on the image at this step to separate the pulmonary nodules attached to the lung wall from the blood vessels. The erosion operator reduces the bright areas of the image and makes the dark areas appear larger as shown in Figure 6a.
6. Applying closure operation (with a disk of radius 10) [15]: The aim of using this operator is to maintain the nodules connected to the lung wall. This operator can remove small dark spots from the image and connect small bright gaps. The image obtained by applying this operator is shown in Figure 6b.
7. Filling in the small holes within binary mask: In some cases, due to a breach in binary conversion using thresholding, a series of black pixels belong to the background appear in the binary image. These areas, known as holes, may be helpful. Therefore, we must obtain these areas by filling them as shown in Figure 6c.

**Figure 7:**
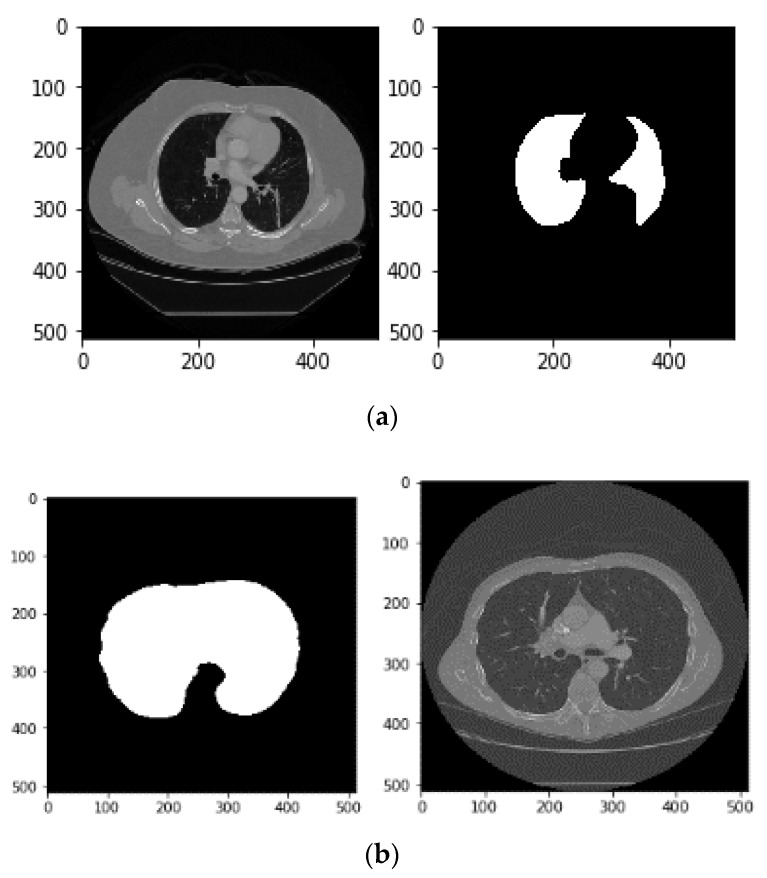
(**a**). Sample of missing a part of the lung in the generated mask, due to considering only the two largest areas, (**b**). Sample of misplaced pixels connecting that fills the non-pulmonary space with white pixels.

In the final step, binary masks are produced which are stored in ‘.bmp’ format. The proposed steps sometimes fail and do not produce the correct binary mask due to two main reasons: (1) all the above steps may cause partial tissues, which could involve lung components, to be ignored in CT scan; (2) sometimes a closure operation, which connects small bright cracks, causes connection of two pixels that fill the non-pulmonary tissue, e.g., air instead of the lung. Figure 7 shows 2 samples of these problems.

Motivated by the above discussions, we need to provide a manual segmentation after producing binary masks by the mentioned algorithm, if necessary. We extracted 1714 binary masks for 10 patients (averagely 170 samples for each patient) using this semi-automatic method. It takes hours to label each CT image by experts, while production of each mask takes on average around three minutes in our proposed method, considering the worst conditions and the need for manual reform. Therefore, the main advantage of this method is to save a lot of time. Also, we plan to publicize our produced masks soon to help other researchers using them in future researches.

### 3.3. Data Preparation

Following the GT extraction described above, we now aim to prepare input raw images to improve the training process of the deep learning network by applying a few preprocessing steps. Therefore, we use two stages including edge detection functions and dilation morphological operations.

**Figure 8:**
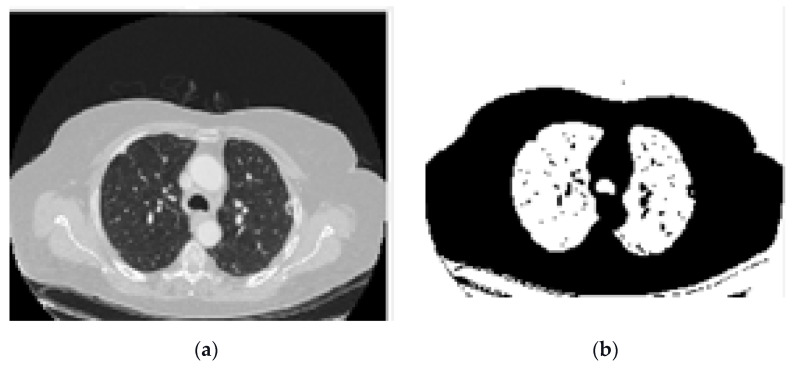
Image binarization process. (**a**). Original CT, (**b**). binarized.

According to the description of the LIDC-IDRI database in previous sections, all CT scans have 512 × 512 resolution and three channels. In this stage of the proposed method, we want to improve the overall segmentation performance. It seems that if we increase the focus of the network during training on a series of specific image features, it will help to improve the forecast. In this regard, we have changed the channels of each image. To do this, we convert these default channels for each CT image to three newly designed channels as follows. In this regard, we use several preprocessing operations such as edge detection functions and dilation morphological operations to generate new images. Then, these images are fed to our proposed network. The main advantage of this idea is that if these newly generated images are fed to a deep neural network, its training can be faster and more accurate. In other words, the proposed channels can provide focused information for the deep neural network which are compatible with the associated masks. This leads to more efficient training and ultimately reduction of false-positive measures. Details of the proposed image conversion are as follows:

a) Image binarization: In this process, a binary image is created with two values on the grey surface, i.e., black and white. The lung region poses a black colour with the value zero. Figure 8 shows the binarization process of a CT image.
b) Dilation morphological operation: Morphological operations, typically applied to binary images, are used to extract and describe the geometry of the object in the image [49,50]. As a result of the binarization process described before, there would still be remaining regions of white colour around the lungs regarded as unwanted noise. Thus, morphological operations can be used to remove these regions. Moreover, there could still be some small black holes in the lung’s region, suspicious of noise caused by the binarization process. These holes should be also removed using morphological operations.

**Figure 9:**
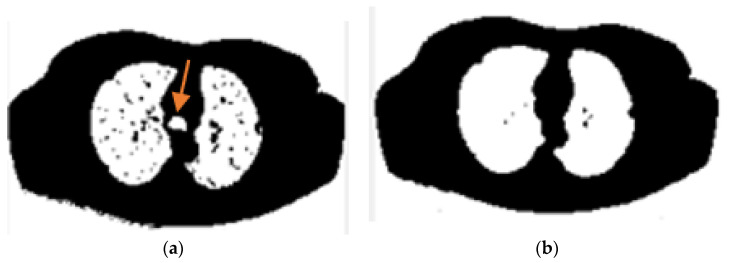
Image after (**a**). binarization; (**b**). dilation.

The morphological operation involves two basic operators: dilation and erosion. Dilation [51] is applied when the segmented object loses part of its target area. This operator increases the target area of the segmentation. It also increases the sensitivity but decreases the specificity. The dilation operation can be mathematically represented as Equation (2).

(2) $$A⊕B=\underset{x\in B}{\cup }A_{x}$$

where A is the image and B is the structuring element. In fact, Equation (1) means that the matrix A is transmitted by each of the points B and then the assembly of all the transferred matrices is calculated. We applied a dilation operation to remove redundant white regions around the lung and small black gaps inside it. Figure 9 shows the result of the dilation process. As can be seen, the orange arrow section (noise) in a binary image is removed in the dilation result.

**Figure 10:**
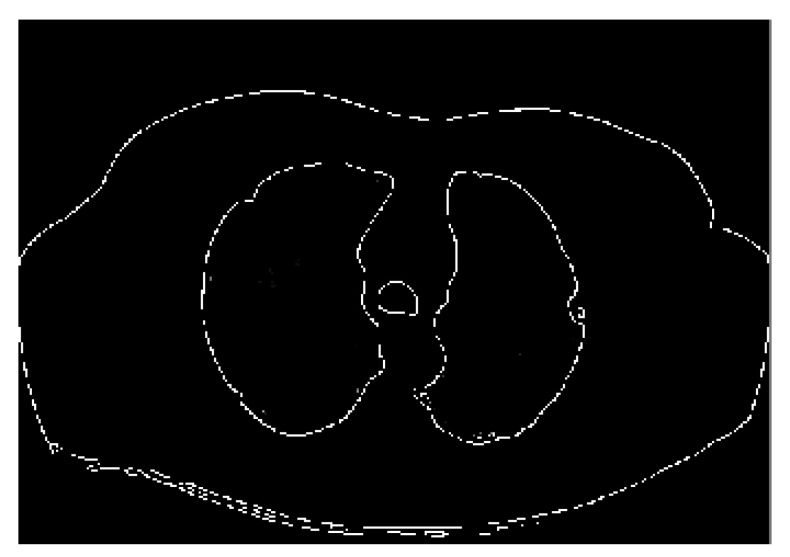
Edge detection using Canny.

c) Edge detection: As already stated, the edge detection filter determines the vertices of an object and the boundaries between objects and the background in the image. This process can also be used to improve the image and eliminate blur. An important advantage of the Canny technique is that it tries to remove the noise of an image before edge extraction and then applies the tendency to find the edges and the critical value of the threshold. Motivated by the advantages expressed so far, we also applied the Canny method to detect the edges in the source images. Figure 10 shows the result of the edge detection process.

**Figure 11:**
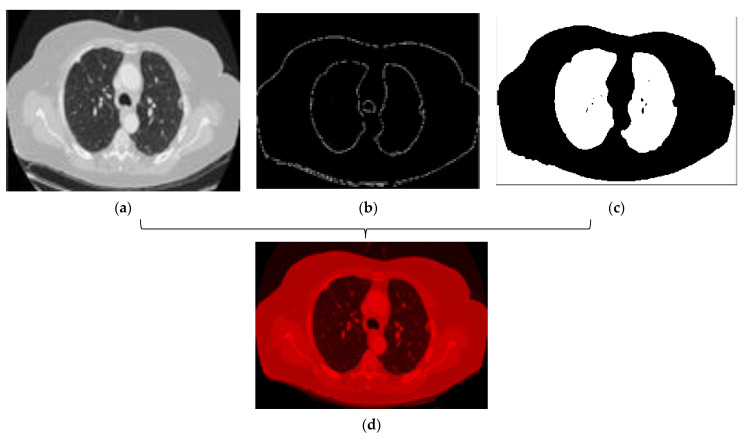
(**a**). First new channel, (**b**). Second new channel, (**c**). The second new channel (**d**). Result of a combination of new channels.

As a result, it cuts down the data quantity and removes unwanted parts, while preserving the required structural features in the image. Next, we need to generate new images with proposed filled channels. The first image channel is filled with the original image (Figure 11a). The second channel of the output image would be an image containing an edge detection process (Figure 11b). In the end, the third new channel would be the image result of the dilation operation (Figure 11c). This helps to reduce the area around the object and also removes the noise. Figure 11 shows the result of the combination of channels. We generated 1714 new lung CT images for 10 patients using the above processing method.

As shown in Figure 11, the resulting image of the combination of the three channels is red. This is due to the arrangement of these channels. As mentioned earlier, the first channel of the new image contains the original image. The second and third channels have been replaced with edge detection processes and dilation operation, respectively. Since black pixels are dominant in the input image (including the edges and resulting image after applying the expansion operations) the final composite image receives the greatest effect from the first channel, leading to a dominant red color. However, if the main image is placed on the second channel, the output image will be green, and similarly blue for the third channel.

### 3.4. Lung Segmentation Using Deep Learning

**Figure 12:**
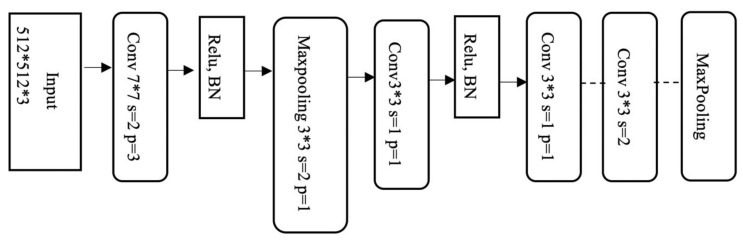
Block diagram of the ResNet-34 in the encoder of Res BCDU-Net.

**Figure 13:**
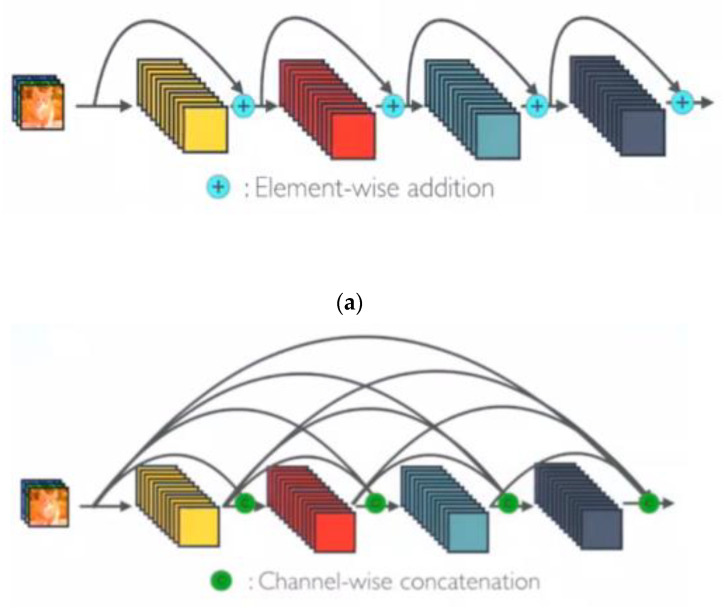
(**a**). ResNet Concept, (**b**). One Dense Block in Dense Net [53].

Since the main goal of this paper is to extract lungs from CT images, our proposed model must successfully address the semantic segmentation problem. U-Net is the most related available deep architecture in this regard. U-Net can learn from a relatively small-size training dataset. In addition, it vastly speeds up training time if a pre-trained model is used. Hence, a good starting point to train the network when dealing with image inputs is using a pre-trained ImageNet model along with its weights. On the other hand, ResBlocks architecture, which was proposed in [47,51], can facilitate the training process, while it offers a deeper network due to having all accumulated layers. Moreover, according to the experiments conducted in different networks and comparing their results, the use of the convolution layer instead of the pooling layer is preferred. This is because pooling layers generate huge semantic feature loss in the image. Thus, it seems ResNet architecture can be a more appropriate choice for the encoder part of the U-Net (the left half of the U). Figure 12 shows the block diagram of the ResNet-34 algorithm used in the encoder section of our proposed network. Our proposed model is mainly inspired by BCDU-Net and ResNet-34 [52] named as Res BCDU-Net. The backbone of this network is a ResNet-34 structure as the encoder which is shown in Figure 13. Details of different layers in the proposed model are described as follows.

**Figure 14:**
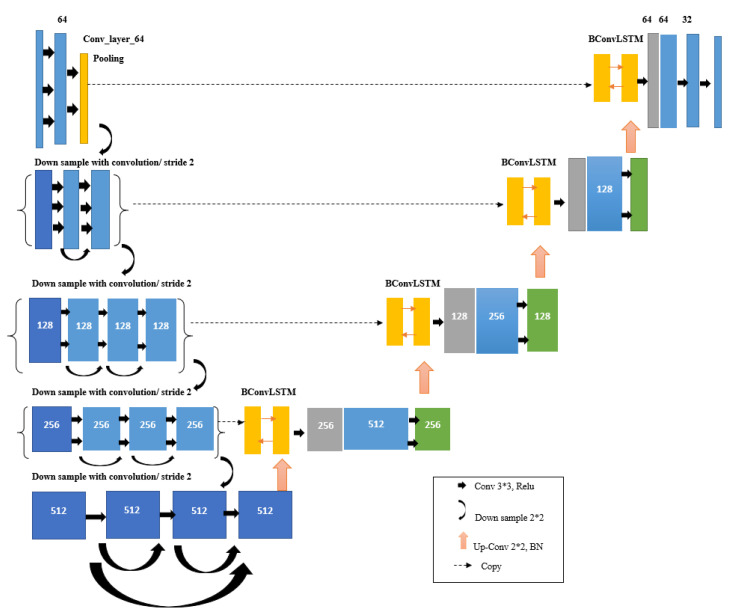
Res BCDU-Net architecture. The contraction path consists of Res blocks and a max-pooling layer. Such the U-Net, in each downsampling of encoding path, feature channels are doubled (64 to 128 to 256 to 512). In the last layer of the contracting path, we used 3 convolutional blocks with 2 dense connections. As seen, in the expansion path, the output of each batch normalized is given to a BConvLSTM layer.

- Encoding path: In Res BCDU-Net, the encoder is replaced with a pre-trained ResNet-34 network. The last layer of this path like BCDU-Net adopts a densely connected convolutions mechanism. So, the last layer, in contrast to all residual blocks in this path, never attempts to combine features through summation before being transferred to a layer; instead, it tries to concatenate the features. In other words, features that are learned per block are passed to the next block. This strategy can help the network to avoid learning redundant features. Figure 13 shows the difference between Res blocks and dense blocks.
- Decoding path: In the decoding path, two feature maps should be concatenated: the feature maps corresponding to the same layer from the encoding path and those from the previous layer of the up-sampling function. In this Network, batch normalization was performed after the output of each up-sampling, before processing of two feature maps. Afterward, the resulting output is given to a BConvLSTM layer. In a standard ConvLSTM, only forward dependencies are processed. However, it is very important not to lose information concealed in any sequence. Therefore, the analysis of both forward and backward approaches has been proven to improve predictive network performance [54]. Both forward and backward ConvLSTMs are considered as standard processes. Therefore, two set parameters are considered as BConvLSTM. This layer can decide on the present input by verifying the data dependencies in both directions. Figure 14 illustrates our proposed network schematically.

## 4. Experimental Results

We evaluated the performance of our proposed neural network on 1714 CT images of the LIDC-IDRI dataset with the corresponding generated ground truth as described in the previous section. The experiments were implemented based on the Keras module with the TenserFlow backend. The network was trained for 50 epochs and batch size 32.

### 4.1. Evaluation Metrics

Several well-established criteria were used for performance evaluation of our proposed network, namely accuracy (AC), precision (Pr), recall (Re), and F1-score. We first calculated true positive (TP), false positive (FP), true negative (TN), and false negative (FN). These performance measures are mathematically expressed as follows:

(3) $$\mathrm{Accuracy}=\frac{\mathrm{TP}+\mathrm{TN}}{\mathrm{TP}+\mathrm{TN}+\mathrm{FP}+\mathrm{FN}}$$

(4) $$\mathrm{Precision}=\frac{\mathrm{TP}}{\mathrm{TP}+\mathrm{FP}}$$

(5) $$\mathrm{Recall}=\frac{\mathrm{TP}}{\mathrm{TP}+\mathrm{FN}}$$

(6) $$F1-\mathrm{score}=2\;\times \frac{\mathrm{Precision}\;\times \;\mathrm{Recall}}{\mathrm{Precision}\;+\;\mathrm{Recall}\;}$$

To turn the results into a more reliable form, Dice’s coefficient [55] is also used to evaluate our results. The Dice score is normally used to determine the performance of the segmentation step on the given images. This is a kind of similarity measure between two different objects. It is equal to the number of overlapping pixels between the two partitions divided by the size of the whole two objects. The Dice score is calculated as:

(7) $$DSC=2\times \frac{\left|E\cap Q\right|}{\left|E|+|Q\right|}$$

where, E is the segmented lung parenchyma area’s pixels based on our network, *Q* is the ground truth image’s pixels and |E∩Q| represents the intersect pixels of two images. We also calculated the receiver operating characteristics (ROC) curve and the area under the curve (AUC). ROC curve is defined as a plot of TPR to FPR, with TPR placed on the *y*-axis and FPR on the *x*-axis. AUC is defined as the underlying area of the ROC curve. In other words, it measures the quality in which the network can segment the input data.

### 4.2. Results

We grouped randomly the dataset into training data (1200 images), validation data (257 images), and test data (257 images) in proportion 70%, 15%, and 15%. We also repeated our experiments 10 times and reported the obtained average performance across all run in this paper. All image sizes are 512 × 512. The input of the network consists of the CT images with three separate designed channels and corresponding ground truth annotations that we generated semi-automatically. Since the image segmentation process corresponds to a pixel-wise classification problem, the task of the neural network is to assign a label or class to all pixels of the input image. The output of the trained network is a pixel-wise mask of the image. Each pixel is given one of two categories:

Class 1: Pixels that fall within the lung area are labelled by ‘0’.
Class 2: Pixels related to the non-lung class are represented by the label ‘1’.

**Figure 15:**
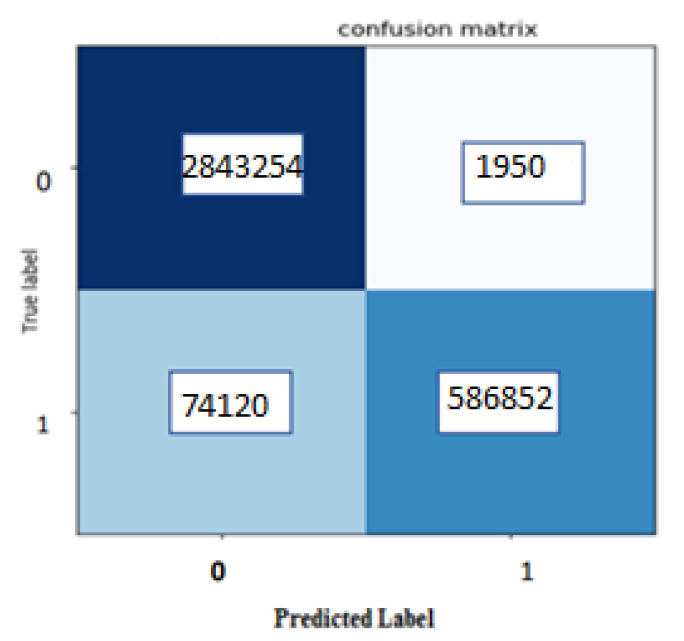
Confusion Matrix for the proposed method.

According to the above descriptions, first, we calculated the confusion matrix as shown in Figure 15.

According to Figure 14, we can see that the TP is very high, and also the point of attention achieved a very low FP. With respect to these values, calculated amounts for the accuracy, precision, recall, and F1-score measures are obtained as 97.83%, 99.93%, 97.45%, and 98.67%, respectively. Table 1 summarizes the results of the precision, recall, F1-score, accuracy, and dice score for another and our methods with LIDC dataset (The best-maintained metrics are highlighted in bold). We also provided some visual example results in Figure 15 to better compare U-Net and BCDU-Net.

According to Table 1, we find that the performance of our proposed method performed better compared to related methods. According to this table, several results can be concluded as follow:

- Using the ResNet34 structure in the encoder section of the U-Net network has considerably improved the obtained results particularly in the quantity of recall.
- BCDU—Net model generally performs better than the ResNet structure in the contracting path of the U–Net.
- Using ResNet within BCDU-Net has achieved a better DSC similarity score compared to cases where these networks are used individually.
- Using images under our designed channels help to improve the quantitative results in all the evaluation criteria in comparison to using default channels.
- The high level of recall in our proposed model (with three new channels) arises from small FP as shown in the confusion matrix.

**Figure 16:**
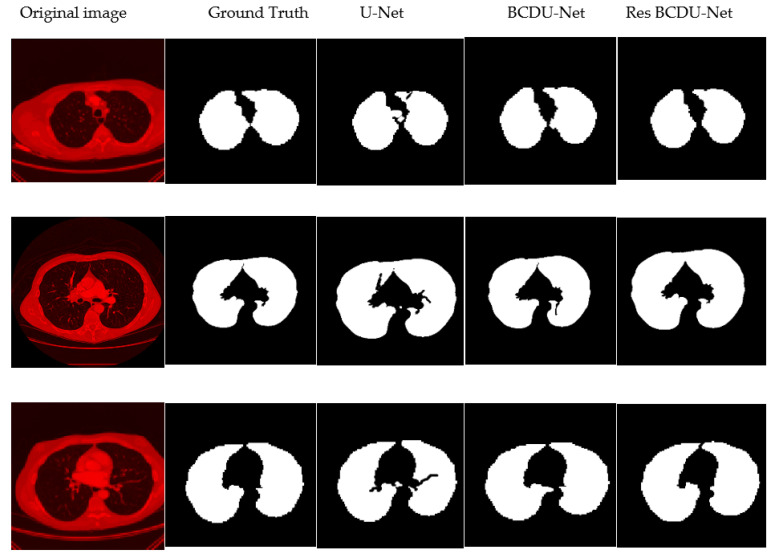
Sample results. From left to right: Original CT image, Ground Truth, U-Net, BCDU-Net, and Proposed method.

As shown in Figure 16, the U-Net model does not work well because of its deficiencies. The BCDU-Net model resolves much of the shortcomings in the image segmentation by U-Net but it sometimes appears a false-positive diagnosis mode (third column). In our proposed method, this problem has been resolved to a large extent and the final segmentation image is much similar to its corresponding mask (compare with U-Net and BCDU-Net in three last columns from right in Figure 16). It can be concluded that the combination of new channels to generate initial CT images and emphasis on components such as the edges and removal of additional items that are irrelevant in new filled channels greatly improves the adaptability power of the network.

**Figure 17:**
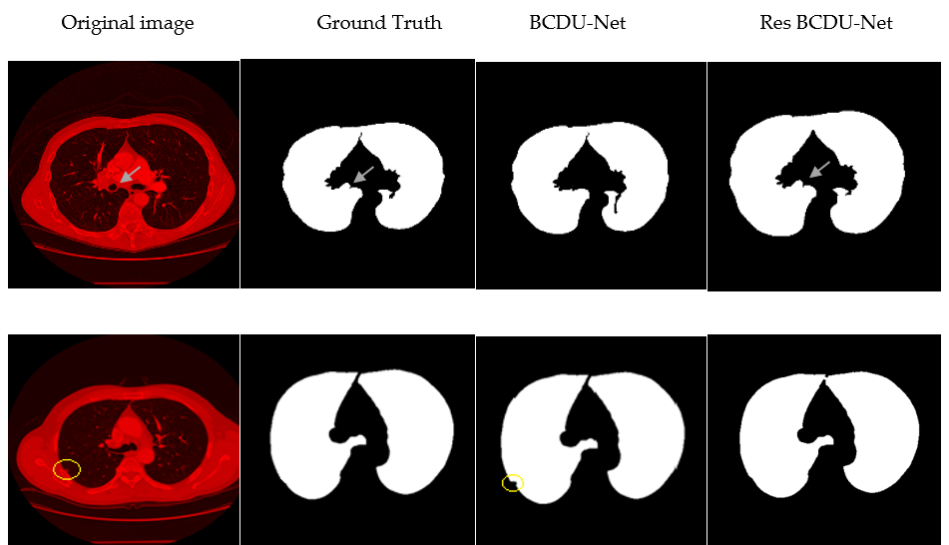
Visualizes the challenges for segmentation. First row presents the challenge of considering micro pulmonary tissues in the segmented image as the non-pulmonary region causing high false positive. Second row presents the challenge of losing attached nodules to the lung wall. (A yellow circle wrapped around the center of the nodule).

The proposed method has solved the high false-positive challenge as well. Also, losing the attached nodules to the lung wall challenge has been resolved by our proposed method (see and compare two last columns from right in Figure 16). It seems that the first challenge is resolved by the idea of combining three new channels in the CT images because it focuses on some components such as the edges and also removes the irrelevant objects and noise in the raw CT images. It can help the final segmentation network to be accurate. The second challenge is resolved by using the ResNet architecture in the first half of BCDU-Net because there is only one Pooling layer in the ResNet architecture and it causes less semantic information to be lost. In addition, the densely connected convolution mechanism in the last layer of the encoding path of the network plays an important role to prevent learning redundant features. To better represent the two above challenges and how the proposed method has resolved them, we have included these two challenges along with the components generated by our algorithm in Figure 17. It seems in this figure, the two challenges described, with the help of our proposed method, are solved using the new hybrid channels in the images and the use of ResNet34 architecture in the encoder section of the neural network.

**Figure 18:**
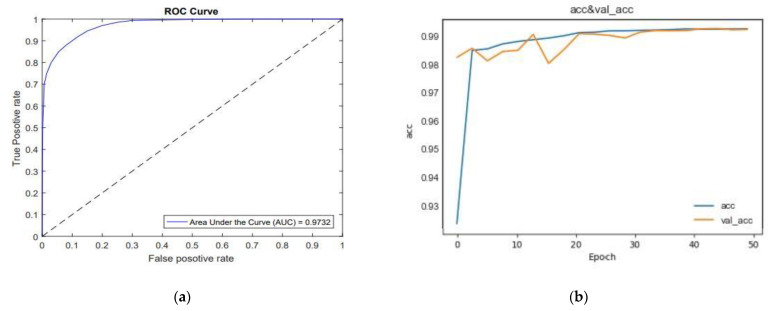
(**a**). ROC curve of Res BCDU-Net; (**b**). The accuracy of training and test for Res BCDU-Net.

The overall performance of our proposed method, the ROC curve and also the accuracy of training and validation proposed network for LIDC-IDRI dataset are shown in Figure 18.

According to Figure 18a, the AUC corresponds to 0.9732 which implies the effectiveness of the proposed model performance. Figure 18b shows that the network converges quickly; on the other hand, it converges after the 35th epoch. We also can see that the accuracy of training increases to over 99% after the 35th epochs. This is a good indicator of appropriate training of the network. In the validation phase, from epoch 0 to 30, it has a descending trend, which indicates inappropriate selection of weights, but the accuracy has been gradually increased from the 35th to 55th epochs. The training and validation accuracy will overlap between 35th and 50th.

### 4.3. Ablation Study

In this section, we conduct the ablation study to determine the effects of each component on the performance of the segmentation system. In detail, we intend to answer these questions in this section: (1) How does the use of images with the new three channels affect the overall performance of the system? (2) What is the effect of automatically producing binary labels for each of the images? (3) What is the effect on execution time and assisting the medical community? (4) What is the effect of using densely connected convolutions and BConvLSTM in the proposed deep neural network on the final performance of the system?

First, we discover the role of the new CT image channels in the segmentation performance. So, we did our experiments using images with their own default channels. The result can be found in Table 2. As we can see, the performance of our proposed method, where CT images are filled with newly designed channels, is higher than when they are filled with default channels.

**Figure 19:**
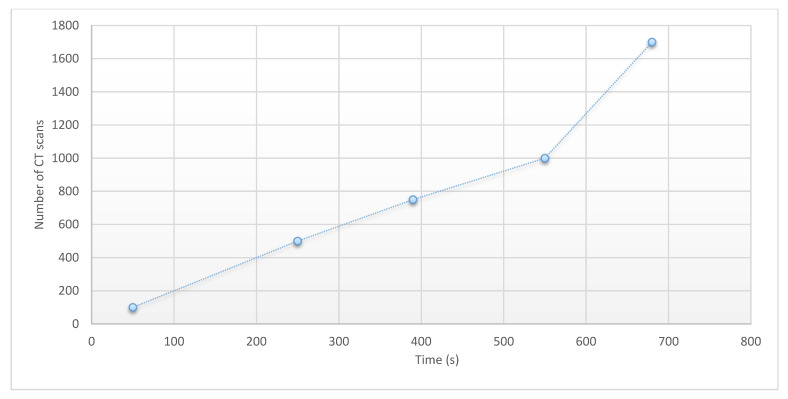
The execution time of the binary mask production algorithm.

As the second work in this section, we look at the running time of the binary mask production algorithm. In this paper, we first used an automated algorithm to produce masks, and then, if necessary, we applied manual modification to each of the generated images. It takes hours to label each CT image taken by the Radiologists; whereas in our proposed method, without manual correction, all masks were produced within 10 min, on average. Considering the worst conditions and the need for manual correction and examination of each image produced by the algorithm, each mask requires 3 min to be made. Looking at Figure 19 the proposed method is capable of producing a similar number of images in a time of nearly 10 min. This figure shows the time of execution measured on the dimension of the data set from 50 to 1700 images. Furthermore, the execution time is reduced to 20% only with respect to the computation time without loading the image. As the number of images increases linearly, we can see that the execution time increases linearly, while the time required for the analysis of images by an expert will be greatly increased by increasing the number of images and parameters such as fatigue and so on.

Finally, we aim to examine the effect of densely connected convolution mechanism in the last layer of the encoding path of neural network and also the rule of using BConvLSTM on the skip connection. Table 3 shows these results. For this comparison, the CT images with new channels are assumed to be the network input, and the ResNet blocks are also used in the encoding section. Given the values in Table 3, we can observe the positive impact of using dense connection mechanism and BConvLSTM on system performance. (Please note that we have already discussed the role of ResNet blocks in the encoding path of the network in Table 1.)

## 5. Conclusions

In this paper, we proposed Res BCDU-Net to automatically and accurately segment the lung region from CT images. The proposed method consists of three main steps. First, we presented a semi-automatic technique to extract the ground truth for each lung. One of the great benefits of our method is that one can manage to produce all mask images, intelligently, without the need for the expertise of a radiologist and that saves a huge amount of time. Second, we proposed a novel three image channel generation and observed a significant decrease in the false positive rate and higher dice coefficients due to effective network input imagery. Finally, we designed the segmentation framework using a novel deep network architecture using a BCDU-Net with an encoder of pre-trained ResNet-34. This model was named Res BCDU-Net. It performed well, as verified through our extensive experiments on the large LIDC-IDRI dataset.

We have seen that combining ResNet and BCDU-Net networks as well as using CT images with newly designed channels in the proposed method has led to a few false positives as well as higher dice similarity scores. We have also seen that by using the automated algorithm used in the label production section for the dataset, the execution time is much less than the one used for producing masks and this is one of the most important advantages of this method.

The application of the proposed algorithm in daily work is being accepted. Because accurate and reliable segmentation of lung tissue is of particular importance in various clinical applications such as computer-assisted bronchoscopy, quantification of emphysema, and diagnosis of lung cancer. Therefore, the great potential goal of our work is applying it to clinical application to help the medical community in their daily work.

## 6. Future Works

One of the interesting research topics that could be pursued in the future is the adaptation and testing of the proposed method for 3D lung CT images. In this regard, a network such as V-Net can be used. Another idea for future works could involve using a combination of deep learning-based networks to segment medical images. It is also possible to examine the use of data enhancement methods and their impact on overall performance.

## Figures and Tables

**Table 1:** Comparison of proposed network performance and the state-of-the-art alternatives on LIDC-IDRI dataset.

Methods	Precision	Recall	F1-Score	Accuracy (%)	Dice Coefficient
U-Net [37]	96.11	96.34	96.22	95.18	95.02
RU-Net [38]	95.52	97.21	96.35	97.15	94.93
ResNet34-Unet [44]	97.32	98.35	97.83	96.73	95.28
BCDU-Net [45]	99.02	98.03	98.52	97.21	96.32
Proposed Method	99.12	97.01	98.05	97.58	97.15

**Table 2:** Impact of CT image channels on system performance.

Channel Type in CT Images	Precision	Recall	F1-Score	Accuracy (%)	Dice Coefficient
Default	99.12	97.01	98.05	97.58	97.15
Proposed	99.93	97.45	98.67	97.83	97.31

**Table 3:** Impact of using densely connected convolutions and BConvLSTM on system performance.

Method	Precision	Recall	F1-Score	Accuracy (%)	Dice Coefficient
Without Densely Connected Convolutions and BConvLSTM	97.02	94.32	95.55	96.21	96.19
Ours (With Densely Connected Convolutions and BConvLSTM)	99.93	97.45	98.67	97.83	97.31

## Data Availability

Not applicable.

## Abbreviations

Acronyms used in the paper.

WHO	World Health Organization
CT	Computed Tomography
MRI	Magnetic Resonance Imaging
CAD	Computer-Aided Diagnosis
BCDU-Net	Bi-directional ConvLSTM U-Net with Densely connected convolutions
FCN	Fully Convolutional Neural Network
CNN	Convolutional Neural Network
BConvLSTM	Bidirectional Convolutional LSTM
LIDC	Lung Image Database Consortium
IDRI	Infectious Disease Research Institute
XML	Extensible Markup Language
DICOM	Digital Imaging and Communications in Medicine
HU	Hounsfield unit
ROC	Receiver Operating Characteristic
AUC	Area under the ROC Curve

## References

[1] Hossain M.R.I., Imran A., Kabir M.H. (2014). Automatic lung tumor detection based on GLCM features. Asian Conference on Computer Vision.

[2] Sun S., Christian B., Reinhard B. (2011). Automated 3-D segmentation of lungs with lung cancer in CT data using a novel robust active shape model approach. IEEE Trans. Med. Imaging.

[3] American Cancer Society‘s Publication, Cancer Facts & Figures 2020. https://www.cancer.org/research/cancer-facts-statistics/all-cancer-facts-figures/cancer-facts-figures-2020.html.

[4] Wang Y., Guo Q., Zhu Y. (2007). Medical image segmentation based on deformable models and its applications. Deformable Models.

[5] Neeraj S., Aggarwal L.M. (2010). Automated medical image segmentation techniques. J. Med. Phys. Assoc. Med. Phys. India.

[6] Asuntha A., Singh N., Srinivasan A. (2016). PSO, genetic optimization and SVM algorithm used for lung cancer detection. J. Chem. Pharm. Res..

[7] Jeyavathana R., Balasubramanian D., Pandian A.A. (2016). A survey: Analysis on preprocessing and segmentation techniques for medical images. Int. J. Res. Sci. Innov..

[8] Panwar H., Gupta P.K., Siddiqui M.K., Morales-Menendez R., Singh V. (2020). Application of deep learning for fast detection of COVID-19 in X-Rays using nCOVnet. Chaos. Solitons. Fractals.

[9] Amine A., Modzelewski R., Li H., Su R. (2020). Multi-task deep learning based CT imaging analysis for COVID-19 pneumonia: Classification and segmentation. Comput. Biol. Med..

[10] Wang X., Deng X., Fu Q., Zhou Q., Feng J., Ma H., Liu W., Zheng C. (2020). A Weakly-supervised Framework for COVID-19 Classification and Lesion Localization from Chest CT. IEEE Trans. Med Imaging.

[11] Hira S., Bai A., Hira S. (2020). An automatic approach based on CNN architecture to detect Covid-19 disease from chest X-ray images. Appl. Intell..

[12] Cheng J., Chen W., Cao Y., Xu Z., Zhang X., Deng L., Zheng C., Zhou J., Shi H., Feng J. (2020). Development and Evaluation of an AI System for COVID-19 Diagnosis. medRxiv.

[13] Pathak Y., Shukla P.K., Tiwari A., Stalin S., Singh S., Shukla P.K. (2020). Deep Transfer Learning based Classification Model for COVID-19 Disease. IRBM.

[14] Rizwan H.I., Neubert J. (2020). Deep learning approaches to biomedical image segmentation. Inform. Med. Unlocked.

[15] Memon N.A., Mirza A.M., Gilani S.A.M. (2006). Segmentation of lungs from CT scan images for early diagnosis of lung cancer. Proc. World Acad. Sci. Eng. Technol..

[16] Omid T., Alirezaie J., Babyn P. Lung segmentation in pulmonary CT images using wavelet transform. Proceedings of the 2007 IEEE International Conference on Acoustics, Speech and Signal Processing-ICASSP’07.

[17] Sasidhar B., Ramesh Babu D.R., Ravi Shankar M., Bhaskar Rao N. (2013). Automated segmentation of lung regions using morphological operators in CT scan. Int. J. Sci. Eng. Res..

[18] Keita N., Shimizu A., Kobatake H., Yakami M., Fujimoto K., Togashi K. (2013). Multi-shape graph cuts with neighbor prior constraints and its application to lung segmentation from a chest CT volume. Med. Image Anal..

[19] Geetanjali J., Kaur S. (2017). A Review on Various Edge Detection Techniques in Distorted Images. Int. J. Adv. Res. Comput. Sci. Softw. Eng..

[20] Shin M.C., Goldgof D.B., Bowyer K.W., Nikiforou S. (2001). Comparison of edge detection algorithms using a structure from motion task. IEEE Trans. Syst. Manand Cybern. Part B Cybern..

[21] Paola C., Casiraghi E., Artioli D. (2006). A fully automated method for lung nodule detection from postero-anterior chest radiographs. IEEE Trans. Med Imaging.

[22] Ana Maria M., da Silva J.A., Campilho A. Automatic delimitation of lung fields on chest radiographs. Proceedings of the 2004 2nd IEEE International Symposium on Biomedical Imaging: Nano to Macro (IEEE Cat No. 04EX821).

[23] Hu X., Alperin N., Levin D.N., Tan K.K., Mengeot M. (1991). Visualization of MR angiographic data with segmentation and volume-rendering techniques. J. Magn. Reson. Imaging.

[24] Tang J., Millington S., Acton S.T., Crandall J., Hurwitz S. (2006). Surface extraction and thickness measurement of the articular cartilage from MR images using directional gradient vector flow snakes. IEEE Trans. Biomed. Eng..

[25] Cline H.E., Dumoulin C.L., Hart H.R., Lorensen W.E., Ludke S. (1987). 3D reconstruction of the brain from magnetic resonance images using a connectivity algorithm. Magn. Reson. Imaging.

[26] Nihad M., Grgic M., Huseinagic H., Males M., Skejic E., Smajlovic M. Automatic CT image segmentation of the lungs with region growing algorithm. Proceedings of the 18th International Conference on Systems, Signals and Image Processing-IWSSIP.

[27] da Silva Felix H.J., Cortez P.C., Holanda M.A., Costa R.C.S. Automatic Segmentation and Measurement of the Lungs in healthy persons and in patients with Chronic Obstructive Pulmonary Disease in CT Images. Proceedings of the IV Latin American Congress on Biomedical Engineering 2007, Bioengineering Solutions for Latin America Health.

[28] Kass M., Witkin A., Terzopoulos D. (1988). Snakes: Active contour models. Int. J. Comput. Vis..

[29] Yoshinori I., Kim H., Ishikawa S., Katsuragawa S., Ishida T., Nakamura K., Yamamoto A. Automatic segmentation of lung areas based on SNAKES and extraction of abnormal areas. Proceedings of the 17th IEEE International Conference on Tools with Artificial Intelligence (ICTAI’05).

[30] Shi Y., Qi F., Xue Z., Chen L., Ito K., Matsuo H., Shen D. (2008). Segmenting lung fields in serial chest radiographs using both population-based and patient-specific shape statistics. IEEE Trans. Med Imaging.

[31] Cheng J., Liu J., Xu Y., Yin F., Wong D.W.K., Tan N.-M., Tao D., Cheng C.-Y., Aung T., Wong T.Y. (2013). Superpixel classification based optic disc and optic cup segmentation for glaucoma screening. IEEE Trans. Med Imaging.

[32] Titinunt K., Han X.-H., Chen Y.-W. Liver segmentation using superpixel-based graph cuts and restricted regions of shape constrains. Proceedings of the 2015 IEEE International Conference on Image Processing (ICIP).

[33] Chen X., Yao L., Zhou T., Dong J., Zhang Y. (2020). Momentum contrastive learning for few-shot COVID-19 diagnosis from chest CT images. arXiv.

[34] Zhou K., Gu Z., Liu W., Luo W., Cheng J., Gao S., Liu J. Multi-cell multi-task convolutional neural networks for diabetic retinopathy grading. Proceedings of the 2018 40th Annual International Conference of the IEEE Engineering in Medicine and Biology Society (EMBC).

[35] Dan C., Giusti A., Gambardella L.M., Schmidhuber J. Deep neural networks segment neuronal membranes in electron microscopy images. Proceedings of the advances in Neural Information Processing Systems.

[36] Jonathan L., Shelhamer E., Darrell T. Fully convolutional networks for semantic segmentation. Proceedings of the IEEE Conference on Computer Vision and Pattern Recognition.

[37] Olaf R., Fischer P., Brox T. U-net: Convolutional networks for biomedical image segmentation. Proceedings of the International Conference on Medical Image Computing and Computer-Assisted Intervention.

[38] Alom M.Z., Yakopcic C., Taha T.M., Asari V.K. Nuclei Segmentation with Recurrent Residual Convolutional Neural Networks based U-Net (R2U-Net). Proceedings of the NAECON 2018—IEEE National Aerospace and Electronics Conference.

[39] Fausto M., Navab N., Seyed-Ahmad A. V-net: Fully convolutional neural networks for volumetric medical image segmentation. Proceedings of the 2016 Fourth International Conference on 3D Vision (3DV).

[40] Özgün C., Abdulkadir A., Lienkamp S.S., Brox T., Ronneberger O. 3D U-Net: Learning dense volumetric segmentation from sparse annotation. Proceedings of the International Conference on Medical Image Computing and Computer-Assisted Intervention.

[41] Zhou X., Ito T., Takayama R., Wang S., Hara T., Fujita H. Three-dimensional CT image segmentation by combining 2D fully convolutional network with 3D majority voting. Proceedings of the Deep Learning and Data Labeling for Medical Applications.

[42] Ozan O., Schlemper J., Folgoc L.L., Lee M., Heinrich M., Misawa K., Mori K., Mori K., McDonagh S., Hammerla N.Y. Attention u-net: Learning where to look for the pancreas. Proceedings of the 1st Conference on Medical Imaging with Deep Learning (MIDL 2018).

[43] Ozsahin I., Sekeroglu B., Musa M.S., Mustapha M.T., Ozsahi D.U. (2020). Review on Diagnosis of COVID-19 from Chest CT Images Using Artificial Intelligence. Comput. Math. Methods Med..

[44] Stephen L., Chong L.H., Edwin K.P., Xu T., Wang X. (2020). Automated Pavement Crack Segmentation Using U-Net-Based Convolutional Neural Network. IEEE Access.

[45] Reza A., Asadi-Aghbolaghi M., Fathy M., Escalera S. Bi-directional ConvLSTM U-net with Densley connected convolutions. Proceedings of the IEEE International Conference on Computer Vision Workshops.

[46] Song H., Wang W., Zhao S., Shen J., Lam K.-M. Pyramid dilated deeper convlstm for video salient object detection. Proceedings of the European Conference on Computer Vision (ECCV).

[47] Christian S., Ioffe S., Vanhoucke V., Alemi A.A. Inception-v4, inception-resnet and the impact of residual connections on learning. Proceedings of the Thirty-First AAAI Conference on Artificial Intelligence.

[48] https://wiki.cancerimagingarchive.net/display/Public/LIDC-IDRI.

[49] Vanitha U., Prabhu Deepak P., PonNageswaran N., Sathappan R. (2015). Tumor detection in brain using morphological image processing. J. Appl. Sci. Eng. Methodol..

[50] Megha G. (2011). Morphological image processing. Int. J. Creat. Res. Thoughts.

[51] He K., Zhang X., Ren S., Sun J. Deep residual learning for image recognition. Proceedings of the IEEE Conference on Computer Vision and Pattern Recognition.

[52] Gao H., Sun Y., Liu Z., Sedra D., Weinberger K.O. Deep networks with stochastic depth. Proceedings of the 14th European Conference on Computer Vision.

[53] Gao H., Zhuang L., Van Der Maaten L., Weinberger K.Q. Densely Connected Convolutional Networks. Proceedings of the 2017 IEEE Conference on Computer Vision and Pattern Recognition (CVPR).

[54] Sayda E. Deep Stacked Residual Neural Network and Bidirectional LSTM for Speed Prediction on Real-life Traffic Data. Proceedings of the 24th European Conference on Artificial Intelligence—ECAI 2020.

[55] Lee D.R. (1945). Measures of the amount of ecologic association between species. Ecology.

